# Exogenous Melatonin Improves Antioxidant Defense in Cucumber Seeds (*Cucumis sativus* L.) Germinated under Chilling Stress

**DOI:** 10.3389/fpls.2016.00575

**Published:** 2016-04-28

**Authors:** Bałabusta Marta, Katarzyna Szafrańska, Małgorzata M. Posmyk

**Affiliations:** Department of Ecophysiology and Plant Development, Faculty of Biology and Environmental Protection, University of LodzLodz, Poland

**Keywords:** antioxidant enzymes, chilling stress, *Cucumis sativus* L., glutathione, hydrogen peroxide, melatonin, seed osmopriming

## Abstract

The relationship between exogenous melatonin applied into cucumber seeds during osmopriming and modifications of their antioxidant defense was studied. Accumulation of hydrogen peroxide, antioxidant enzyme activities and glutathione pool were investigated in embryonic axes isolated from the control, osmoprimed, and osmoprimed with melatonin seeds. Germinating cucumber seeds are very sensitive to chilling. Temperature 10°C causes oxidative stress in young seedlings. Seed pre-treatment with melatonin seemed to limit H_2_O_2_ accumulation during germination under optimal condition as well as during chilling stress and recovery period. Melatonin affected superoxide dismutase (SOD) activity and its isoforms during stress and recovery period but did not influence CAT and POX activities. Thus it is possible that in cucumber this indoleamine could act mostly as a direct H_2_O_2_ scavenger, but superoxide anion combat *via* SOD stimulation. The GSH/GSSG ratio is considered as an indirect determinant of oxidative stress. When the cells are exposed to oxidative stress GSSG is accumulated and the ratio of GSH to GSSG decreases. In our research pre-sowing melatonin application into the cucumber seeds caused high beneficial value of GSH/GSSG ratio that could be helpful for stress countering. Glutathione reductase (GSSG-R) activity in the axes isolated from these seeds was two fold higher than in those isolated from the control and from the osmoprimed without melatonin ones. Additional isoforms of GSSG-R in melatonin treated seeds were also observed. It explains high and effective GSH pool restoration in the seeds pre-treated with melatonin. We confirmed that melatonin could protect cucumber seeds and young seedlings against oxidative stress directly and indirectly detoxifying ROS, thereby plants grown better even in harmful environmental conditions. This work is the first that investigated on plant *in vivo* model and documented melatonin influence on redox state during seed germination. This way we try to fill lack of information about melatonin-regulated pathways involved in antioxidant strategy of plant defense.

## Introduction

A very important aspect of modern crop production is to prevent environmental stress during the growing season. In addition to overcome various effects of different types of stresses (temperatures, water deficit, xenobiotic pollutions, etc.) very important for the preferred/optimal homeostasis is to maintain an appropriate redox status of plant cells. It is known that oxidative stress is the secondary one in relation to all environmental stress faced by plant. Especially critical for plants are the periods of: germination and initial growth of young seedlings, than flowering and fruit development. In this paper we presented an effective method to counteract temperature stress in the early life stage of chilling-sensitive plant – *Cucumis sativus* L.

Pre-sowing seed treatments effectively counteract diseases and pests as well as improve seed vigor and seedlings viability. Knowledge concerning physiology, biochemistry and anatomy of plants is crucial for application of these techniques. The main principle of priming methods is controlled hydration of seeds and their subsequent re-drying to initial water content for further potential storage. There are three main types of seed conditioning: (i) water soaking (hydropriming), (ii) soaking in low water potential solutions (osmopriming), (iii) treatment with solid matrices (solid matrix-priming). These processes lead to imbibition of seeds thus preparing them for sowing. Priming increases activities of antioxidant enzymes, intensifies protein and nucleic acid metabolism and facilitates cell membrane structure reorganization (Siadat et al., 2012; Jisha et al., 2013). These techniques can be also combined with other supporting methods like aeration, pelleting but also application of bioactive substances i.a. fungicides, hormones, biostimulators, into the seeds (Jisha et al., 2013). Our research group was the first that applied melatonin into the seeds as exogenous biostimulator (Posmyk et al., 2008, 2009a,c; Janas et al., 2009; Szafrańska et al., 2012, 2013, 2014; Kołodziejczyk et al., 2015).

Melatonin (*N*-acetyl-5-methoxytryptamine) is a highly conserved molecule occurring in evolutionary distant organisms. It has been described in bacteria, monocellular and multicellular algae, higher plants, vertebrates and invertebrates (Hardeland, 2016; Nawaz et al., 2016). For many years melatonin was considered only as a hormone and neurotransmitter in animals, but now its occurrence in plants is well documented (Posmyk and Janas, 2009; Arnao, 2014; Arnao and Hernandez-Ruiz, 2015). In the plant kingdom it was discovered in 1991 in autotrophic unicellular dinoflagellates *Lingulodinium polyedrum* Stein (formerly *Gonyaulax polyedra*), now belonging to *Protista* (Balzer and Hardeland, 1991). Since then, research concerning its presence and possible functions in other plants has been intensified. Investigations are based on the assumption that the role of melatonin in plants is similar to that in animals. In both cases tryptophan is the basic precursor of indoleamine compounds, including melatonin (Hardeland, 2016). According to new findings melatonin plays several important functions in plants. It may act as a regulator of growth and plant development (bears a strong resemblance to plant auxin – IAA) (Arnao and Hernández-Ruiz, 2006; Nawaz et al., 2016), a circadian cycle regulator (hormone of darkness) (Kolář and Macháčková, 2005), as well as it may influence cell division and control mitotic spindle formation (Murch and Saxena, 2002). It was also observed that plants accumulate high levels of melatonin when faced with harsh environments (Arnao and Hernández-Ruiz, 2014; Zhang et al., 2015) and exogenously applied melatonin helps improve tolerance to stresses (Janas and Posmyk, 2013; Kołodziejczyk and Posmyk, 2016).

Melatonin is ubiquitously present in organisms whose metabolism is based on oxygen, so it is speculated that its primary function is to protect organisms against oxygen toxicity. It is known to be an endogenous free radical scavenger and a broad-spectrum antioxidant. It detoxifies a variety of free radicals and reactive oxygen species (ROS) including hydroxyl radical (OH^•^), peroxynitrite anion (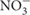), singlet oxygen (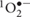) and nitric oxide (NO^•^) (Tan et al., 2007). One of the most appealing properties of this molecule, which distinguishes it from most antioxidants, is the fact that its metabolites also have the ability to scavenge ROS and reactive nitrogen species (RNS). Melatonin generates free radical scavenging cascade providing continuous cell defense, which makes this molecule, even at low concentrations, highly effective in protecting organisms from oxidative stress (Galano et al., 2013). Although melatonin acts as a direct antioxidant it may also regulate activities of several antioxidant enzymes including superoxide dismutase (SOD), catalase (CAT), ascorbate peroxidase (APX), glutathione peroxidase (GSH-PX), glutathione reductase (GSSG-R), as it was described in animal tissues (Rodriguez et al., 2004; Fischer et al., 2013). ROS are generated in response to unfavorable environmental conditions like chilling stress. Consequences of ROS production depend on the intensity of stress, which regulates the amount of ROS and physiological and chemical cell condition. The active oxygen produced under stress is a harmful factor, which causes lipid peroxidation, enzyme inactivation and DNA damage. On the other hand, the crucial role of ROS in plant signal transduction is also known. Thus plant’s dilemma is not how to totally eliminate ROS, but how to control them (Posmyk, 2009; Considine et al., 2015).

Organisms have evolved defense mechanisms to protect cells from the damage caused by ROS which efficiently and rapidly remove excess of ROS from intracellular space: (i) antioxidant enzymes scavenging ROS (e.g., SOD, CAT), (ii) low molecular mass antioxidants (e.g., glutathione, ascorbate, phenolic compounds, carotenoids, tocopherols) directly or in cooperation with enzymes (peroxidases – POX) scavenging free radicals, (iii) enzymes needed to regenerate the active forms of antioxidants (e.g., GSSG-R, monodehydro- and dehydro-ascorbate reductases – MDHA-R and DHA-R) (Posmyk, 2009). When the equilibrium between free radicals and the antioxidant defense system is imbalanced in favor of the oxidants, oxidative stress appears. The oxidants that are not scavenged or metabolized may attack cellular components producing thereby useless molecular debris, which sometimes leads to cell death (Rodriguez et al., 2004).

Antioxidant enzymes, which metabolize ROS to innocuous byproducts are the first line of defense against their toxicity (Rodriguez et al., 2004; Posmyk, 2009). ROS might be eliminated by protective mechanisms involving free radical- and peroxide-scavenging enzymes such as SOD, CAT, GSSG-R and POX. SOD can be considered as a key enzyme within the defense mechanisms against oxidative stress, determining the cellular concentration of 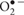 and H_2_O_2_. It converts 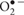 radical into H_2_O_2_ and then CAT is implicated in converting H_2_O_2_ into water and O_2_. Similarly to CAT, GSSG-R and POX could also be engaged in the control of endogenous H_2_O_2_ level through an oxide-reduction cycle involving glutathione and ascorbate or phenolics (Posmyk et al., 2009b). POX decomposes H_2_O_2_ by oxidation of different co-substrates such as phenolic compounds and other proton donors. POXs that use glutathione as a co-substrate have only rarely been observed (Rao et al., 1996).

Glutathione participates in both enzymatic and non-enzymatic H_2_O_2_ degradation. The GSH/GSSG ratio is considered as a determinant of oxidative stress. When the cells are exposed to oxidative stress GSSG is accumulated and the ratio of GSH to GSSG decreases. GSSG-R is the key enzyme for GSH pool restitution.

Increased expression (different isoforms) and activity of antioxidant enzymes in cells exposed to oxidative stress improve their protection from the damage caused by ROS. Sometimes the number of free radicals may be so great that even growing activity of the antioxidant enzymes is insufficient to counteract the potential damage. When plants are exposed to intense oxidative stress the expression and activity of antioxidant enzymes can decrease due to damage of the molecular machinery, which is required to induce enzymatic protection. However, a moderate level of toxic reactants can induce increased activity of antioxidant enzymes (Considine et al., 2015).

Our previous works with different plants (*Brassica oleracea*, *Cucumis sativus*, *Zea mays*, and *Vigna radiata*) showed that melatonin applied to the seeds had positive influence on their germination as well as on the development of seedlings especially under environmental stresses (Posmyk et al., 2008, 2009a; Janas et al., 2009; Szafrańska et al., 2012, 2013, 2014; Kołodziejczyk et al., 2015). The purpose of the presented work was to study how exogenous melatonin can affect redox status of cells and if it can modify antioxidant enzyme activities in cucumber (*Cucumis sativus* L. var Odys) embryonic axes isolated from seeds pretreated with melatonin. Comparison was made between differently primed seeds variants: control (C) – untreated, osmoprimed (O), osmoprimed with melatonin: 50 and 500 μM water solutions (OMel50, OMel500 respectively). There were three experimental procedures of germination for each seed variant (i) optimal conditions at 25°C, (ii) chilling stress at 10°C, (iii) chilling stress with subsequent recovery at 25°C.

## Materials and Methods

### Plant Material

Cucumber seeds (*Cucumis sativus* L. var. Odys) were provided by TORSEED (Torun, Poland). They were stored in the dark, under dry conditions, at room temperature, in tightly closed containers before the experiments started.

### Osmopriming

The seeds were osmoprimed (O) according to the methods described previously by Posmyk et al. (2001). They were placed for 5 days on a layer of cotton wool containing PEG-8000 solution at -1.5 MPa. The osmotic potential of PEG was calculated according to Michel and Kaufman (1973). PEG was dissolved in distilled water (O) or melatonin solutions to final concentrations of 50 and 500 μM (OMel50, OMel500, respectively). The treatments were carried out in plastic rectangular boxes (18 cm long, 12 cm wide and 5.5 cm deep) with 100 seeds per box, placed at 25°C in darkness.

### Seeds Germination Conditions

The seed variants (C, O, OMel50, OMel500) placed on 9-cm diameter Petri dishes (30 seeds per dish) with two layers of Whatman 2 filter paper (Whatman International Ltd. Maidstone, UK) wetted with distilled water were germinated: (i) at optimal temperature 25°C for 24 h (t_0_), (ii) at chilling stress temperature 10°C for 7 days (CS7), (iii) at 10°C for 7 days and then they were recovered at optimal 25°C for 2 days. Embryonic axes isolated from seeds prepared as mentioned above were used for biochemical tests.

### H_2_O_2_ Determination

The H_2_O_2_ content in cucumber axes was determined according to Sergiev et al. (1997). Fresh tissues (1 g_FW_) were ground in a mortar with a pestle in 5 ml of 0.1 % TCA. The homogenate was centrifuged at 20000 *g* for 15 min at 4°C. Supernatant (0.5 ml) was added to the reaction medium containing 0.5 ml 10 mM phosphate buffer and 1 ml 1 M KI. Absorbance was determined at 390 nm by a UV-Vis spectrophotometer (Hitachi U-2001). The results were estimated from H_2_O_2_ calibration curve, expressed as μmol per gram fresh weight [μmol g_FW_^-1^] – mean values of nine measurements ± SEM.

### Glutathione Pool Estimation

The cucumber axes (0.3 g_FW_) were homogenized with ice-cold 5% TCA containing 0.2 mM EDTA. After centrifugation at 20000 *g* for 15 min at 4°C, the supernatant (0.4 ml) was neutralized with 0.6 ml 0.5 M phosphate buffer pH 7.5. For the oxidized glutathion (GSSG) assay, reduced glutathion (GSH) was masked by adding 30 μl of 2-vinylpiridyne to the neutralized supernatant, whereas 30 μl of water was added for the total glutathione (GT) pool assay. The tubes were shaked until an emulsion was formed. Glutathione content was measured in 2 ml of the reaction mixture containing 0.02 mM NADPH, 100 mM phosphate buffer (pH 7.5), 5 mM EDTA, 0.6 mM DTNB and the extract. The reaction was started by adding 6 units of GSSG-R and was monitored by measuring the changes in absorbance at 412 nm. GSH was estimated as the differences between the amount of GT and GSSG (GSH = GT–2GSSG) (Tybulski and Tretyn, 2010). The results were expressed as nmol per gram fresh weight [nmol g_FW_^-1^] – mean values of nine measurements ± SEM.

### Enzyme Extraction and Assay

One gram of fresh weigh of axes isolated from the control or osmoprimed seeds was ground in a mortar and homogenized with 0.5 g PVP in 5 ml of 0.1 M phosphate buffer (pH 7.5) containing 2.5 mM DTT, 1 mM EDTA, 1.25 mM PEG-4000 and 1 mM PMSF. The homogenate was centrifuged at 20000 *g* for 30 min at 4°C. The resulting supernatant was filtered through Miracloth, desalted on a PD10 column (Pharmacia, Uppsala, Sweden) and used for the enzyme assays. All steps of the extraction procedure were carried out at 4°C.

#### Superoxide Dismutase

Superoxide dismutase (EC 1.15.1.1) activity was measured according to Giannopolitis and Ries (1977). The reaction mixture contained 2 μM riboflavine, 13 mM methionine, 0.1 mM EDTA, 70 μM NBT in 0.1 M phosphate buffer (pH 7.5), and 100 μl of the enzyme extract in the final volume of 3 ml. SOD activity was assayed by measuring the ability of the enzyme extract to inhibit the photochemical reduction of NBT. Glass test tubes containing the mixture were illuminated with a fluorescent lamp at 25°C (Philips MLL 5000 W, Eindhoven, Netherlands). Identical tubes, which were not illuminated served as blanks. After illumination for 15 min, absorbance was measured at 560 nm. One unit of SOD was defined as the enzyme activity, which inhibited the photoreduction of NBT to blue formazan by 50%. SOD activity was expressed as the enzyme unit per miligram of protein [U mg_prot_^-1^].

#### Catalase

Catalase (EC 1.11.1.6.) activity was measured at 25°C according to Clarbone (1985). The enzyme assay mixture contained 18 mM H_2_O_2_ in 0.1 M phosphate buffer (pH 7.0) and 100 μl of the enzyme extract in the total volume of 2 ml. CAT activity was estimated by the decrease in absorbance of H_2_O_2_ at 240 nm and was expressed as micromoles of H_2_O_2_ decomposed during 1 min per 1 mg of protein [μmol min^-1^ mg_prot_^-1^].

#### Peroxidase

Peroxidase (EC 1.11.1.7) activity was determined at 25°C according to Scebba et al. (2001). The reaction mixture contained 2.25 mM guaiacol, 11 mM H_2_O_2_ in 0.1 M phosphate buffer (pH 6.0), and 100 μl of the enzyme extract in the total volume of 2 ml. POX activity was determined by following the increase in absorbance of guaiacol at 470 nm, and was expressed as micromoles of guaiacol oxidized during 1 min per 1 mg of proteins [μmol min^-1^ mg_prot_^-1^].

#### Glutathione Reductase

Glutathione reductase (EC 1.6.4.2) activity was determined at 30°C according to Esterbauer and Grill (1978), by following the rate of NADPH oxidation at 340 nm. The assay mixture contained 0.5 mM NADPH, 10 mM GSSG, 6.25 mM MgCl_2_ in 0.1 M phosphate buffer (pH 7.5), and 100 μl of the enzyme extract in the total volume of 400 μl. GSSG-R activity was expressed as micromoles of NADPH oxidized during 1 min per 1 mg of proteins [μmol min^-1^ mg_prot_^-1^].

SOD, CAT, POX, and GSSG-R activities of each extract were measured three times, and the presented results correspond to the means ± SEM of the values obtained with three different extracts (*n* = 9). Protein content of the extracts was determined according to the method of Bradford (1976).

### Preparation of Protein Extracts and Native-PAGE

Protein extraction for native PAGE was similar to previous described in Section “Enzyme Extraction and Assay.” To increase the protein concentration in the extracts and for protein purification, the supernatants were concentrated on AMICON ULTRA-0.5 ml 10 kDa ultracel-PL membrane – centrifugal filters (MILLIPORE, UFC501096) at 20000 *g* for 30 min at 10°C. Protein content of the extracts was determined according to the method of Bradford (1976).

#### Native-PAGE

The samples of tissue were suspended in the loading buffer (0.5 M Tris-HCl buffer pH 6.8, glycerol, 0.5% (w/v) bromophenol blue). Protein extracts were subjected to PAGE under non-denaturing and non-reducing conditions as described by Laemmli (1970), except that SDS was omitted. The portions of 20/40 μg of protein per line were separated on 12/8%, respectively (4% stacking gel) during 16 h at 10°C with a constant voltage of 20 V per gel using Mini-Protean Tetra cell (BioRad, Hertz, UK).

#### GSSG-R Staining

Glutathione reductase was separated on 8% polyacrylamide gel. GSSG-R activity staining gel was performed by 30-min incubation in darkness at 25°C in a solution containing 150 mM Tris-HCl buffer pH 8.0, 3 mM EDTA, 3.4 mM oxidized glutathione, 3.4 mM NADPH, 0.2 mg ml^-1^ of 2,6-dichlorophenolindophenol (DCPIP) and 0.2 mg ml^-1^ of 3-(4,5-dimethyl-2-thiazolyl)-2,5-dophenyl-2H-tetrazolium bromide (MTT) (Rao et al., 1996). The gel was rinsed with distilled water after staining.

#### SOD Staining

Superoxide dismutase was separated on 12% polyacrylamide gel. Isoenzymes of SOD were detected on the gel by the method of Beauchamp and Fridovich (1971) with modifications. Gel was incubated for 25 min in the light (light is necessary for photoreaction in the SOD activity staining) in 50 mM sodium phosphate buffer pH 7.8 with 2.45 mM of nitrobluetetrazolium (NBT). Next the gel was placed in a 50 mM sodium phosphate buffer pH 7.8 containing 28 μM riboflavin and 28 μM TEMED. After staining the gel was rinsed with distilled water.

#### Densitometric Analysis of the Native Gels

Isoforms were detected and their activity was estimated by densitometric analysis of the strips in native gels using QuantityOne 1-D Analysis Software v. 4.6.9 (BioRad). Activity of individual isoforms was calculated relative to a standard solution of the proper enzyme: SOD from Bovine Liver (Sigma S8409) or GSSG-R from Bakers Yeast (Sigma G3664), with regard to the background of the gel.

### Statystical Analysis

The data were analyzed by two-way analysis of variance (ANOVA) and then the *post hoc* Duncan’s multiple range tests was carried out to find the significant differences at *p* < 0.01 in the particular experiments and variants.

## Results

**FIGURE 1:**
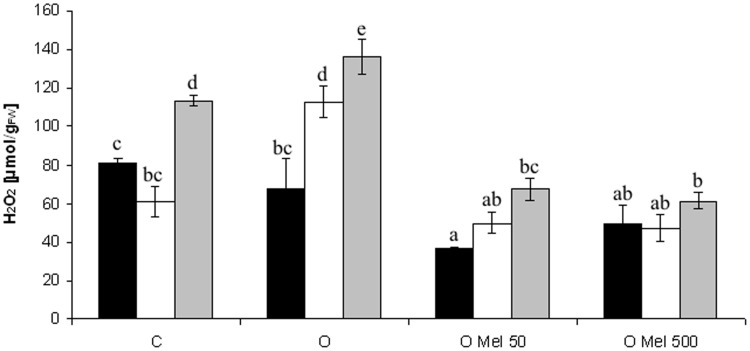
**Effect of cucumber seed osmopriming variants on hydrogen peroxide content in the axes isolated from seeds: control - untreated (C), osmoprimed without (O) and with melatonin at concentrations: 50 μM (OMel50) and 500 μM (OMel500).** The seeds were imbibed/germinated in water at 10°C in darkness for 7 days (CS7 – □), and then subsequently recovered at 25°C for 2 days (R2 – 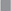). Hydrogen peroxide content was also estimated in the axes isolated from the seeds germinated for 24 h under optimal conditions 25°C (to –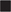). The results are expressed as mean values of nine measurements ± SEM. Two-way ANOVA and Duncan’s *post hoc* test were performed. The small letters next to the values show statistical significance *p* < 0.001. H_2_O_2_ ANOVA results: Variant (C, O, OMel50, OMel500) *F*(3.82) = 51.3, *p* < 0.001, Crucial Point (to, CS7, R2) *F*(2.82) = 36.6, *p* < 0.001 and interaction Variant × Crucial Point *F*(6.82) = 6.3, *p* < 0.001.

Cucumber seeds are very sensitive to low temperature, but research of Posmyk et al. (2008) showed that osmopriming with melatonin significantly increased their tolerance to chilling. In the presented work temperature of 10°C caused oxidative stress in germinating seeds, which resulted in ROS generation (**Figure 1**). Hydrogen peroxide (H_2_O_2_) is the most common ROS and its level increases when plants are subjected to chilling stress. However, the best way to estimate the oxidative damages – that are less visible in low temperature – is the recovery of stressed plants at optimal conditions. In the axes isolated from the control (C) and osmoprimed without melatonin (O) seeds significant increase in H_2_O_2_ level was observed, especially after 2 days of recovery (**Figure 1**). For comparison, the seeds osmoprimed with melatonin OMel50 and OMel500 exhibited the lowest H_2_O_2_ accumulation during the experiment. Even if under chilling stress (10°C) and shortly afterward the increase in H_2_O_2_ content in OMel50 seeds was noted (**Figure 1**), finally the amount of H_2_O_2_ in these seeds was twofold lower then in the C and O ones. The lowest H_2_O_2_ content was observed in OMel500 seeds, which remained at the same low level during the whole experiment.

**FIGURE 2:**
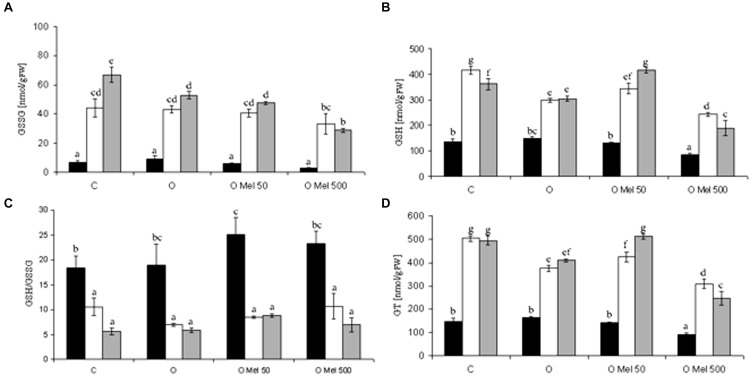
**Effect of cucumber seed osmopriming variants on glutathione pool: GSSG – oxidized glutathione **(A)**, GSH – reduced glutathione **(B)**, GSH/GSSG ratio **(C)**, GT – total glutathione **(D)** in the axes isolated from seeds: control - untreated **(C)**, osmoprimed without (O) and with melatonin at concentrations: 50 μM (OMel50) and 500 μM (OMel500).** The seeds were imbibed/germinated in water at 10°C in darkness for 7 days (CS7– □), and then subsequently recovered at 25°C for 2 days (R2 – 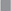). Glutathione pool was also estimated in the axes isolated from the seeds germinated for 24 h in optimal conditions 25C (to –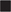). The results are expressed as mean values of nine measurements ± SEM. Two-way ANOVA and Duncan’s *post hoc* test were performed. The small letters next to the values show statistical significance *p* < 0.001. GSSG ANOVA results **(A)**: Variant (C, O, OMel50, OMel500) F(3.56) = 12.8, *p* < 0.001, Crucial Point (to, CS7, R2) *F*(2.56) = 152.4, *p* < 0.001 and interaction Variant × Crucial Point *F*(6.56) = 4.3, *p* < 0.001. GSH ANOVA results **(B)**: Variant (C, O, OMel50, OMel500) *F*(3.57) = 45.56, *p* < 0.001, Crucial Point (to, CS7, R2) *F*(2.57) = 214.55, *p* < 0.001 and interaction Variant × Crucial Point *F*(6.57) = 9.5, *p* < 0.001. GSH/GSSG ANOVA results **(C)**: Variant (C, O, OMel50, OMel500) *F*(3.54) = 1.77, *p* < 0.05, Crucial Point (to, CS7, R2) *F*(2.54) = 53.55, *p* < 0.001 and interaction Variant × Crucial Point *F*(6.54) = 0.823, *p* < 0.05. GT ANOVA results **(D)**: Variant (C, O, OMel50, OMel500) *F*(3.59) = 65.3, *p* < 0.001, Crucial Point (to, CS7, R2) F(2.59) = 403.6, *p* < 0.001 and interaction Variant ^∗^ Crucial Point *F*(6.59) = 12.3, *p* < 0.001.

GSH can directly detoxify superoxide and hydroxyl radical and thus contribute to non-enzymatic ROS scavenging (Noctor et al., 2012). The GSH/GSSG ratio reflects the intensity of oxidative stress. During germination at 25°C total glutathione (GT) contents were similar in all experimental variants (**Figure 2D**). Its level increased under chilling stress condition and remained high after 2-days of recovery. Its lowest amount was observed in OMel500 seedlings (**Figure 2D**). Similar changes occurred in GSSG content (**Figure 2A**). Glutathione oxidation increased under chilling stress and still remained high after the recovery period, but simultaneously the greatest rise of GSH fraction was noticed in the control and OMel50 seeds (**Figure 2B**). Finally, however, the most beneficial GSH/GSSG ratio was observed before stress in the seeds primed with melatonin (OMel50 and OMel500) (**Figure 2C**).

**FIGURE 3:**
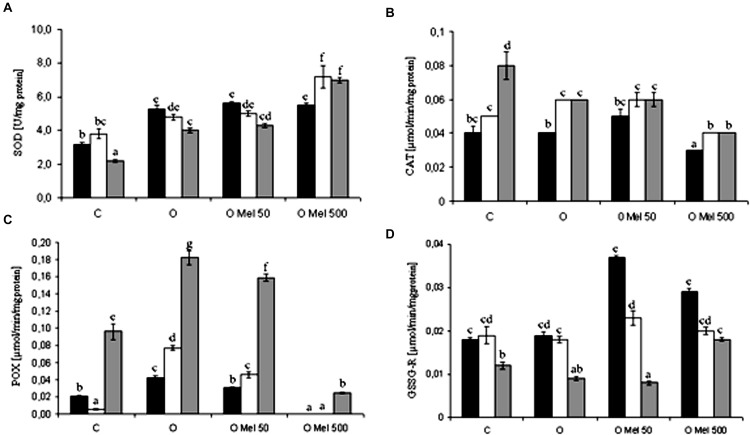
**Effect of cucumber seed osmopriming variants on different antioxidant enzymes activity: SOD - superoxide dismutase **(A)**, CAT – catalase **(B)**, POX – peroxidase **(C)**, GSSG-R – glutathione reductase **(D)** in the axes isolated from seeds: control – untreated (C), osmoprimed without (O) and with melatonin at concentrations: 50 μM (OMel50) and 500 μM (OMel500).** The seeds were imbibed/germinated in water at 10°C in darkness for 7 days (CS7 – □), and then subsequently recovered at 25°C for 2 days (R2 – 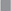). Enzymes activity was also estimated in the axes isolated from the seeds germinated for 24 h in optimal conditions 25°C (to –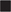). The results are expressed as mean values of nine measurements ± SEM. Two-way ANOVA and Duncan’s *post hoc* test were performed. The small letters next to the values show statistical significance *p* < 0.001. SOD ANOVA results **(A)**: Variant (C, O, OMel50, OMel500) *F*(3.60) = 101.4, *p* < 0.001, Crucial Point (to, CS7, R2) *F*(2.60) = 10.8, *p* < 0.001 and interaction Variant × Crucial Point *F*(6.60) = 9.2, *p* < 0.001. CAT ANOVA results **(B)**: Variant (C, O, OMel50, OMel500) *F*(3.60) = 44.6, *p* < 0.001, Crucial Point (to, CS7, R2) *F*(2.60) = 59.2, *p* < 0.001 and interaction Variant × Crucial Point *F*(6.60) = 4.4, *p* < 0.001. POX ANOVA results **(C)**: Variant (C, O, OMel50, OMel500) *F*(3.58) = 255, *p* < 0.001, Crucial Point (to, CS7, R2) *F*(2.58) = 547.6, *p* < 0.001 and interaction Variant × Crucial Point *F*(6.58) = 42.3, *p* < 0.001. GSSG-R ANOVA results **(F)**: Variant (C, O, OMel50, OMel500) *F*(3.57) = 49.4, *p* < 0.001, Crucial Point (to, CS7, R2) *F*(2.57) = 238.3, *p* < 0.001 and interaction Variant × Crucial Point *F*(6.57) = 38.1, *p* < 0.001.

Enzymatic and non-enzymatic antioxidant systems are directly or indirectly responsible for ROS removing from cells. Various antioxidant enzymes are considered as major antioxidants. SOD is a transition metal-containing enzyme which catalyzes dismutation of the superoxide anion (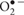) to molecular oxygen and hydrogen peroxide and thus it is a crucial part of the cellular antioxidant defense mechanism. After 24 h of germination at 25°C SOD showed higher activity in all osmoprimed seeds as compared to the control ones (**Figure 3A**). However, under 7 days of chilling stress and after 2 days of recovery, slight decrease in SOD activity was observed, except OMel500 where significant increase in its activity was noted (**Figure 3A**).

Chilling stress in all experimental variants in a similar way stimulated CAT. Its lowest activity was observed in OMel500 seeds while the highest in the control seeds after the recovery period (**Figure 3B**).

Under chilling stress in the embryo axes isolated from O and OMel50 seeds POX activity increased significantly (**Figure 3C**). It was especially high after the recovery period in all seed variants except OMel500 where it reached only about 10% of the highest value in O seeds (**Figure 3C**).

GSSG-R is an antioxidant enzyme highly sensitive to the chilling stress. Its activity was twofold higher in OMel50 and OMel500 in comparison to the C and O seeds germinating under optimal condition, but chilling stress decreased its activity in all seed variants (**Figure 3D**). At chilling temperature the highest activity of GSSG-R was observed in OMel50 seeds (**Figure 3D**).

**FIGURE 4:**
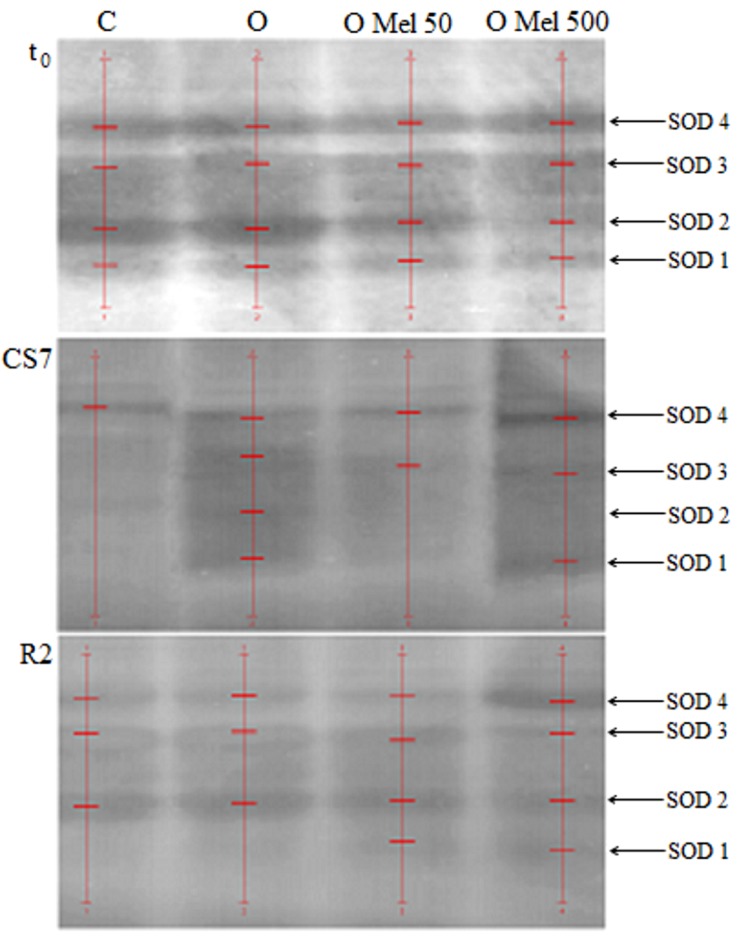
**Native gels with superoxide dismutase (SOD) isoenzymes extracted from embryonic axes of the cucumber seeds.** The axes were isolated from control seeds (C) and those osmoprimed without (O) and with melatonin at concentrations: 50 μM (O Mel 50) and 500 μM (O Mel 500). The seeds were germinated in water at 10°C in darkness for 7 days (CS7), and then subsequently regenerated at 25°C for 2 days (R2). Enzymes were also estimated in the axes isolated from the seeds germinated 24 h in optimal conditions 25°C (t_0_). The stripes of enzyme isoforms were detected (red lines) and estimated by densitometric analysis of gels using QuantityOne 1-D Analysis Software v. 4.6.9 (BioRad). In the case of SOD native gels their original image – dark gels with light strips – ware automatically transformed to their negative versions by software.

Since SOD and GSSG-R seem to depend on exogenous melatonin application to the seeds during osmopriming additional analyses were performed to monitor changes in the activity of potential isoforms of these enzymes in all experimental variants. As shown in **Figure 4**, four isoforms of SOD were determined in native gels. SOD isoform activities estimated by densitometric analysis of these gels are collected in **Table 1**. Under all experimental conditions the highest SOD activity was obtained for OMel500 seeds where the most remarkable activity was recorded at SOD 4 isoform. In the other seed variants there was no such trend and, e.g., in OMel50 axes at optimal conditions and after recovery period SOD 2 isoform showed the maximum activity, while under chilling stress it was not noticeable and SOD 4 isoform was the most active. In the control seeds increased activity was exhibited by SOD 1 isoform, whereas after chilling and recovery it was not detectable. Finally, these results obtained in native gels are consistent with the SOD activity measured spectrophotometrically in biochemical tests (**Table 1**; **Figure 3A**).

**Table 1:** Superoxide dismutase (SOD) isoform activities estimated by densitometric analysis of the native gels (QuantityOne - BioRad).

Seed variant	SOD isoform	SOD [U]		
		
		t_0_	CS7	R2
C	SOD 4	0.99	6.62	1.18
	SOD 3	0.95	-	0.73
	SOD 2	0.78	-	2.07
	SOD 1	2.76	-	-
	
	Σ	5.48	6.62	3.98

O	SOD 4	5.61	6.80	1.70
	SOD 3	1.60	2.64	1.53
	SOD 2	6.30	1.67	4.59
	SOD 1	1.54	1.90	-
	
	Σ	15.05	13.01	7.82

O Mel 50	SOD 4	2.44	8.92	2.12
	SOD 3	2.22	5.73	2.58
	SOD 2	9.59	-	4.50
	SOD 1	0.99	-	0.45
	
	Σ	15.24	14.65	9.65

O Mel 500	SOD 4	9.59	9.78	13.43
	SOD 3	5.48	7.52	1.08
	SOD 2	2.44	-	7.40
	SOD 1	3.39	7.11	0.52
	
	Σ	20.9	24.41	22.43


*The activity of individual isoforms was estimated relative to a standard solution of the enzyme with regard to the background of the gel. Analyses were performed for all variants of the seeds: control (C), osmoprimed without (O) and with melatonin at concentrations: 50 μM (OMel50) and 500 μM (OMel500). The seeds were imbibed/germinated in water at 10°C in darkness for 7 days (CS7), and then subsequently recovered at 25°C for 2 days (R2). Enzymes activity was also estimated in the axes isolated from the seeds germinated for 24 h in optimal conditions 25°C (t_0_).*

**FIGURE 5:**
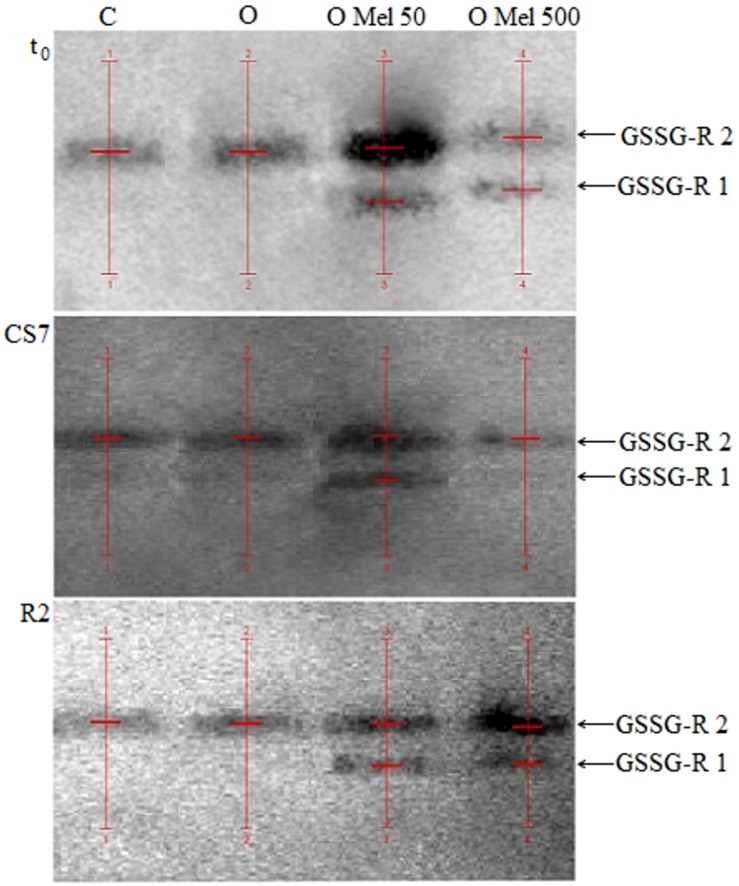
**Native gels with glutathione reductase (GSSG-R) isoenzymes extracted from embryonic axes of the cucumber seeds.** The axes were isolated from control seeds (C) and those osmoprimed without (O) and with melatonin in concentration: 50 μM (O Mel 50) and 500 μM (O Mel 500). The seeds were germinated in water at 10°C in darkness for 7 days (CS7), and then subsequently recovered at 25°C for 2 days (R2). Enzymes were also estimated in the axes isolated from the seeds germinated 24 h in optimal conditions 25°C (t_0_). The stripes of enzyme isoforms were detected (red lines) and estimated by densitometric analysis of gels using QuantityOne 1-D Analysis Software v. 4.6.9 (BioRad).

To check GSSG-R isoform activities native gels for this enzyme were prepared (**Figure 5**). According to **Figure 5** and data in **Table 2** only in the axes isolated from the seeds pretreated with melatonin (OMel50, OMel500) two isoforms of GSSG-R were detected (GSSG-R 1, GSSG-R 2). In the C and O seeds in all experimental variants only GSSG-R 2 isoform activity was detected. Even if the activity of GSSG-R 2 isoform was comparable in all seed variants, the final, total activity of this enzyme doubled in OMel50 and OMel500 due to the appearance of GSSG-R 1 isoform (**Table 2**). Additionally, contrary to biochemical test, in the native gels significant decrease in GSSG-R activity after recovery period was not observed (**Table 2**; **Figure 3D**).

**Table 2:** GSSG-R isoform activities estimated by densitometric analysis of the native gels (QuantityOne – BioRad).

Seed variant	GSSG-R isoform	GSSG-R [U]		
		
		t_0_	CS7	R2
C	GSSG-R 2	6.57	6.57	6.61
	GSSG-R 1	-	-	-
	
	Σ	6.57	6.57	6.61

O	GSSG-R 2	6.55	6.60	6.62
	GSSG-R 1	-	-	-
	
	Σ	6.55	6.60	6.62

O Mel 50	GSSG-R 2	6.61	6.62	6.62
	GSSG-R 1	6.49	6.58	6.60
	
	Σ	13.10	13.20	13.22

O Mel 500	GSSG-R 2	6.60	6.56	6.62
	GSSG-R 1	6.56	-	6.59
	
	Σ	13.16	6.56	13.21


*The activity of individual isoforms was estimated relative to a standard solution of the enzyme with regard to the background of the gel. Analyses were performed for all variants of the seeds: control (C), osmoprimed without (O) and with melatonin at concentrations: 50 μM (OMel50) and 500 μM (OMel500). The seeds were imbibed/germinated in water at 10°C in darkness for 7 days (CS7), and then subsequently recovered at 25°C for 2 days (R2). Enzymes activity was also estimated in the axes isolated from the seeds germinated for 24 h in optimal conditions 25°C (t_0_).*

## Discussion

Because melatonin is a non-toxic substance of natural origin that improves and stimulates plant life it is considered as a biostimulator (Janas and Posmyk, 2013; Arnao and Hernandez-Ruiz, 2015; Kołodziejczyk and Posmyk, 2016). Endogenous melatonin concentrations differ not only from species to species but also among varieties of the same species. It was detected and quantified in roots, shoots, leaves, flowers, fruits, and seeds, but its highest level was found in reproductive organs, particularly in seeds (Paredes et al., 2009; Posmyk and Janas, 2009; Janas and Posmyk, 2013). It was suggested that variations in melatonin contents might result from different environmental impact during plant growth and development as well as during consecutive stages of seed morphological and physiological development. An increase in melatonin content was detected in sunflower seeds during sprouting (Cho et al., 2008). Since the germ tissue is highly vulnerable to oxidative damage, melatonin might be present as an important component of its antioxidant defense system as a free radical scavenger in a dormant and more or less dry system, where enzymes are poorly effective and cannot be up regulated. Thus, melatonin in seeds may be essential for protecting plant germ and reproductive tissues from oxidative injuries (Manchester et al., 2000). The physiological concentration of melatonin in some seeds is very high, for example in white and black mustard seeds it reaches 129 and 189 ng g^-1^, respectively. This content is much higher than its physiological concentration in blood of many vertebrates (Nawaz et al., 2016).

It was observed that various plant species rich in MEL have shown higher capacity for stress tolerance (Park et al., 2013; Bajwa et al., 2014; Zhang et al., 2015). Melatonin is also involved in stress-affected developmental transitions including flowering, fruiting, ripening and senescence (Kolář and Macháčková, 2005; Arnao and Hernández-Ruiz, 2009; Zhao et al., 2013; Byeon and Back, 2014).

Since it is possible that melatonin plays an important role in plant stress defense it was natural to develop a simple and inexpensive method for increasing its content in plants. Our previous works indicated that exogenous melatonin application into seeds by osmo- and hydropriming is a very effective method improving its content and such application significantly increases the positive effects of seed priming. It is suitable for cucumber seed osmopriming (Posmyk et al., 2009a,b) as well as sweet corn (Janas et al., 2009; Kołodziejczyk et al., 2015) red cabbage (Posmyk et al., 2008); and *Vigna radiata* (Szafrańska et al., 2013, 2014) seed hydropriming. Priming combined with melatonin application protects membrane structures against peroxidation and protein oxidation during chilling stress and recovery (Posmyk et al., 2008; Szafrańska et al., 2013, 2014).

It was suggested that melatonin as an indoleamine structurally similar to IAA could play a role similar to that of auxins (Nawaz et al., 2016), even if mechanisms of their action are different (Pelagio-Flores et al., 2012). However, it seems that evolutionary the strong antioxidant properties of melatonin were its primary role in the plant stresses tolerance and defense against unfavorable conditions, e.g., extreme heat, cold, pollution and UV radiation (Peyrot and Ducrocq, 2008; Galano et al., 2013).

Various stresses inhibit plant growth *via* different mechanisms but all cause rises in ROS production and disturb red-ox homeostasis. It is well known that oxidative stress is a secondary effect of all biotic and some abiotic ones. Since melatonin is soluble in both water and lipids, it may be a hydrophilic and hydrophobic antioxidant. This fact together with melatonin small size makes it particularly able to migrate easily between cell compartments in order to protect them against excessive ROS. Thus, melatonin is a broad-spectrum antioxidant. Moreover, recent evidence indicates that the primary melatonin metabolites, especially N1-acetyl-N2-formyl-5-methoxykynuramine (AFMK) also have high antioxidant abilities. The AFMK could be formed by numerous free-radical reactions thus a single melatonin molecule is reported to scavenge up to 10 ROS. It is documented that the free radical scavenging capacity of melatonin extends to its secondary, tertiary and quaternary metabolites (Tan et al., 2007; Galano et al., 2013). This process is referred to as the free radical scavenging cascade, which makes melatonin even at low concentrations highly effective at protecting organisms against oxidative stress. This cascade reaction is characteristic of melatonin making it more efficient than other conventional antioxidants.

Cucumber (*Cucumis sativus* L.) is very sensitive to low temperatures; optimal temperature for its germination is 20–25°C, no germination is observed below 10°C. Melatonin application significantly increased its resistance to this abiotic stress (Posmyk et al., 2009a,b) but the mechanism of this phenomenon is still not clear.

H_2_O_2_ is a ROS, which is formed when oxygen accepts two electrons. H_2_O_2_ is positioned at the center of the oxygen radical generating chain. Its metabolism determines formation of highly toxic species, however, at the physiological concentration it is not toxic. In the Fenton or Haber–Weiss reactions it is converted to the highly cytotoxic HO^•^. H_2_O_2_ level increased in rice cultivars after plant exposure to chilling stress (Guo et al., 2006). The same results were obtained for the control embryo axes isolated from the cucumber seeds after 7-day chilling stress and recovery (**Figure 1**). Exactly the same effect was observed in the O seeds (**Figure 1**). H_2_O_2_ and its derivatives can be very harmful so it is important for plants to develop a strategy to scavenge H_2_O_2_ so as to avoid HO^•^ formation. In OMel50 and OMel500 cucumber seeds the accumulation of H_2_O_2_ was lower in comparison to C and O seeds under all conditions studied (optimal temperature, chilling stress and recovery period) (**Figure 1**). Similarly, high H_2_O_2_ content in wheat seedlings induced by cold stress was markedly reduced by melatonin pre-treatment (Turk et al., 2014). Long-term exogenous application of melatonin delayed also drought-induced leaf senescence in apple in which H_2_O_2_ level was much lower than in non-treated ones (Wang et al., 2013). Thus melatonin can act as a highly effective factor protecting plant cells from oxidative damage. Its protective activity was also observed in the embryo axes isolated from cucumber (Posmyk et al., 2009a,b), corn (Kołodziejczyk et al., 2015), and red cabbage (Posmyk et al., 2008) seeds where melatonin participated in protection of membrane structures against damage caused by chilling stress and recovery.

Abiotic stresses are major limiting factors in crop production. Many injuries are associated with oxidative damage at the cellular level caused by deformation of crucial molecules such as nucleic acids, functional and structural proteins and membrane lipids. Consequences of ROS formation depend on the combination of the following factors: intensity of stress and physicochemical cell conditions, i.e., antioxidant status, integrity of membranes, energy resources and redox state (Posmyk, 2009). Plant cells exposed to oxidative stress can activate their oxygen-scavenging systems on the one hand by increasing the expression and/or activity of antioxidant enzymes, on the other hand by mobilization of their non-enzymatic antioxidants such as reduced glutathione (GSH), ascorbate, tocopherols, carotenoids, or phenolic compounds – as compensatory mechanisms to protect themselves better against damage caused by free radicals.

GSH is one of the major non-enzymatic tissue antioxidants and detoxifying agents additionally involved in xenobiotics removal (Franco and Cidlowski, 2009). It is a critical source of reducing power because it takes part in the reduction of H_2_O_2_ to H_2_O with formation of disulfide bonds between two GSH molecules, that gives rise to GSSG – oxidized glutathion. GSSG-R recycled GSSG to GSH. The central cysteine group in the GSH frame is essential for the regulation of disulfide bonds of proteins and in the disposal of electrophiles and oxidants. This makes GSH a very important cell molecule, but its action as a cellular redox buffer is still poorly understood if we take into account its low redox potential and occurrence at millimolar concentration in cells (Franco and Cidlowski, 2009). During prolonged oxidative stress GSSG is accumulated and the GSH/GSSG ratio decreases. In the presented research pre-sowing application of melatonin into the cucumber seeds resulted in high beneficial value of GSH/GSSG ratio (**Figure 2C**) that could be helpful for eventual future stress combating. In the seeds treated with melatonin also the accumulation of H_2_O_2_ was much lower than in both embryo axes isolated from the C and O seeds. Elevated level of GSH is correlated with environmental stress tolerance, therefore enhanced GSH synthesis appears to be an intrinsic plant response to stress (Noctor et al., 2012). These results indicate that melatonin may stimulate GSH synthesis. This is consistent with studies of Turk et al. (2014) in which foliar application of melatonin to wheat seedlings exposed to cold stress enhanced total GSH content, as well as markedly increased the ratio of GSH/GSSG compared to the control and cold-stressed without melatonin plants. A similar melatonin influence on total glutathione content was shown by Wang et al. (2012) during dark-induced senescence of detached apple leaves treated with 10 mM of this indoleamine. The beneficial effects of melatonin are frequently associated with attenuation of oxidative damage (Galano et al., 2013). In addition to direct ROS scavenging it also affects the activity of antioxidant enzymes, which was first published in the mid 1990s. These papers described the amplification of GSH-POX and SOD activities in brain and several animal tissues after exogenous application of melatonin (Rodriguez et al., 2004). Subsequent years of research on melatonin resulted in several papers describing its impact on the activity of antioxidant enzymes also in plants. The main H_2_O_2_-scavenging enzymes, CAT and POX presented similar trends in their activities, showing significantly higher activities in melatonin-treated apple leaves than in non-treated ones under either well-watered or drought conditions (Wang et al., 2013). On the other hand, as shown by Turk et al. (2014) in wheat seedlings, the activities of SOD, GPX, APX and GR increased under chilling stress, while CAT activity did not change. Pre-treatment with melatonin resulted in rise of these enzyme activities (except CAT) in comparison to seedlings chilled without melatonin. Also in the presented studies beneficial effect of melatonin on CAT activity was not found because under chilling stress it slightly increased in all investigated variants, not only in those treated with melatonin (**Figure 3B**). Therefore it seems that CAT activity does not depend on melatonin action.

Analysing the activity of non-specific POX under chilling stress, its increase was found only in O and OMel50 seeds but during recovery period it was extremely high in all variants of cucumber seeds except OMel500 seeds (**Figure 3C**). Even though Wang et al. (2013) found positive correlation between melatonin and POX activity in apple leaves, according to our results it is difficult to say that this enzyme activity is melatonin dependent and it seems that this molecule does not play a crucial role in its regulation.

On the other hand in the axes of cucumber seeds treated with low temperature SOD activity were induced only in the OMel500 seeds (**Figure 3A**). In the case of other variants it decreased under chilling stress. As shown by Turk et al. (2014) foliar application of melatonin to cold-stressed wheat seedlings also resulted in elevation of SOD activity, which can suggest that this molecule can play an important role in stimulation of enzymatic superoxide anion dismutation. In cucumber axes germinated under optimal conditions an increase in SOD activity were the same both in the seeds osmoprimed without melatonin as well as in those osmoprimed with melatonin addition (**Figure 3A**). The elevation of SOD activity after osmopriming was observed also in the axes isolated from soybean seeds where its activity was higher in comparison to the control ones (Posmyk et al., 2001).

In order to more precisely assess melatonin influence on changes in SOD activity its isozymes were also monitored. As shown in **Figure 4**, in the cucumber axes four isoforms of SOD were observed in the native gels. Cold stress decreased the activity of all SOD isoforms as compared with the control, except OMel500 seeds, in which all SOD isoforms exhibited still high activities, particularly SOD4 and SOD1. These results are consistent with those obtained by Turk et al. (2014) showing that the increase in SOD activity was confirmed by changes in SOD isozymes.

Reiter et al. (1999) suggested that melatonin could significantly increase not only the activity of SOD and GPX, but also of GSSG-R, which is a very important antioxidant enzyme that restores GSH pool. Similar effect was observed in the axes of cucumber seeds pre-treated with melatonin (OMel50 and OMel500), where GSSG-R activity was twofold higher than in those isolated from the C and O seeds (**Figure 3D**). Although this enzyme seems to be chilling sensitive and low temperature decreased its activity in all seeds, in the axes isolated from the seeds pre-treated with melatonin GSSG-R activity was slightly higher than in the control ones. The O seeds exhibited almost the same GSSG-R activity as the control ones (**Figure 3D**). It is interesting that in the axes isolated from OMel50 and OMel500 seeds incubated under optimal conditions very high increase in the activity of this enzyme was noticed. This was confirmed by the results visualized in native gels obtained for GSSG-R isoforms (**Figure 5**). In OMel50 seeds incubated at 25°C extremely high expression of GSSG-R isoforms, especially characteristic of GSSG-R 2, was observed. These gels confirmed also the highest GSSG-R activity in OMel50 axes under chilling stress and in OMel500 ones after recovery period (**Figures 3D** and **5**). Moreover, in the melatonin treated seeds additional GSSG-R 1 isoform was observed (**Figure 5**).

It is a common phenomenon that when the cells are exposed to high oxidative stress the activity of antioxidant enzymes diminishes. Thus it has been proposed that a moderate level of toxic reactants induces antioxidant enzyme activity while their high level reduces it due to damage of molecular machinery that is required to mobilize these enzymes (Rodriguez et al., 2004). This hypothesis may explain to some extent the changes in antioxidant enzyme activities observed in the present study.

Taking into account our results it is likely that antioxidative mechanisms supported by melatonin are more complex than those of classic antioxidants. Melatonin is not only able to scavenge the highly toxic ROS, but to detoxify their precursors as well. As an antioxidant it seems to function *via* a number of ways: (i) as a direct free radical scavenger, (ii) stimulating antioxidant enzymes especially SOD and GSSG-R, (iii) stimulating *de novo* synthesis of glutathione, (iv) augmenting the activities of other antioxidants (GSH/GSSG ratio) (v) protecting antioxidant enzymes from oxidative damage. This capacity makes melatonin a potentially important end very effective antioxidant. The fact that exogenous melatonin applied into the seeds affects not only the activity but also genetic expression of antioxidant enzymes such as SOD and GSSG-R, generating their additional isoforms is the crucial finding of the presented research.

Due to the lack of information more research on classic plant models is necessary to elucidate melatonin role and its mechanisms of action in plants, but today we can say that this indoleamine used as a biostimulator to regulate plant growth has great potential to improve crop yield under unfavorable environmental conditions.

## Author Contributions

MB: all experiments concerning cucumber seeds/seedlings, data acquisition and analysis, drafting of the manuscript. KS: methodological consultant, data analysis, drafting of the manuscript. MP: conception and design of the work, data analysis and interpretation, the manuscript revising and final approval of the version to be published.

## Conflict of Interest Statement

The authors declare that the research was conducted in the absence of any commercial or financial relationships that could be construed as a potential conflict of interest.
